# Primary Small Cell Carcinoma of the Breast: An Approach to Medical and Surgical Management

**DOI:** 10.7759/cureus.47981

**Published:** 2023-10-30

**Authors:** Megan T Frame, Jaskaren Gohal, Kelsey Mader, Judie Goodman

**Affiliations:** 1 Internal Medicine, University of Kentucky, Lexington, USA; 2 Internal Medicine, Trinity Health Oakland/Wayne State University Program, Pontiac, USA; 3 Medicine, Ross University School of Medicine, Pontiac, USA; 4 Hematology and Oncology, Trinity Health IHA Medical Group, Hematology Oncology - Oakland Campus, Pontiac, USA

**Keywords:** small cell carcinoma of breast, extra-pulmonary small cell carcinoma, rare breast cancer, surgical management, radiotherapy, chemotherapy, neuroendocrine carcinoma of breast

## Abstract

Primary small cell carcinoma of the breast (PSCCB) is a rare and aggressive tumor. Due to the small number of PSCCB cases, there are no established treatment protocols. We report a case of a 55-year-old perimenopausal woman who presented with a palpable left breast mass. A breast mass biopsy was performed, and pathology was consistent with poorly differentiated neuroendocrine small cell carcinoma. Imaging with magnetic resonance imaging (MRI) and positron emission tomography (PET) excluded metastatic disease. Due to recurrent positive margins, the patient underwent two lumpectomies and received neoadjuvant chemotherapy in between procedures. She ultimately underwent a left mastectomy and received postoperative radiation therapy. This case report aims to highlight the challenges that ensue following a diagnosis of PSCCB and underscores the need for creating a standardized treatment model for these vulnerable patients.

## Introduction

Primary small cell carcinoma of the breast (PSCCB) is a rare and aggressive tumor, constituting less than 1% of all breast cancers [1]. The lungs are the most common site for small cell carcinoma, but approximately 2-5% can occur in extrapulmonary sites [1, 2]. Extrapulmonary small cell carcinomas occur more commonly in the gastrointestinal and genitourinary tracts but have also been rarely described in the breast, larynx, esophagus, prostate, pancreas, salivary glands, nasal cavity, cervix, and skin [1-3].

The World Health Organization's 2019 (fifth edition) breast cancer classification categorizes breast carcinomas with neuroendocrine features into three groups: well-differentiated neuroendocrine tumors, which architecturally resemble carcinoid tumors of other sites; moderately differentiated (atypical carcinoids); and poorly differentiated neuroendocrine carcinoma or small cell carcinoma, which is identical to pulmonary small cell carcinoma [4, 5]. Due to PSCCB's pathologic similarity to pulmonary small cell carcinoma, it is important to first exclude metastatic disease both clinically and radiologically before making a final diagnosis. Because of the rarity of this type of breast cancer, the limited data available for PSCCB are based primarily on case reports. Therefore, there is no established treatment protocol for the treatment and management of PSCCB.

We report a case involving a 55-year-old perimenopausal woman with locally advanced PSCCB of the left breast who was managed with a multidisciplinary approach. This approach involved consecutive lumpectomies, neoadjuvant chemotherapy, and ultimately a left mastectomy followed by post-mastectomy radiation therapy. This case offers a look into the importance of early and correct diagnosis, an approach to PSCCB management, and the need for continued reporting of PSCCB to develop standardization of treatment for this rare tumor subtype.

## Case presentation

**Figure 1:**
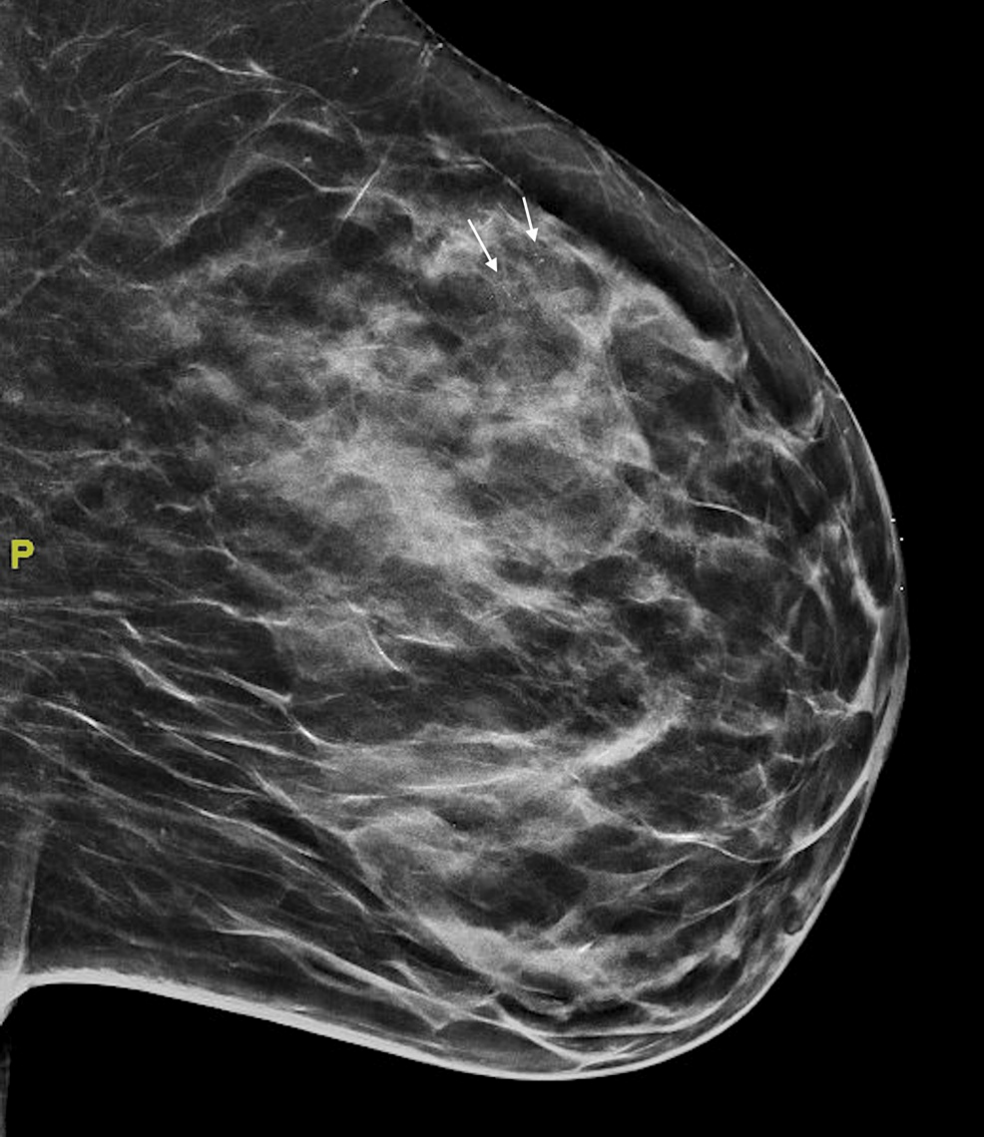
Diagnostic mammogram: left mediolateral oblique view showing left upper outer breast microcalcifications measuring up to 13 mm. This mammogram was performed approximately 16 months prior to the presentation of the breast mass.

A 55-year-old perimenopausal woman presented with a palpable mass in her left breast that she noticed during a self-breast exam. The patient is a life-long non-smoker. Her family history is significant for breast cancer in her maternal grandmother and a maternal cousin with colorectal cancer. Despite her family history, genetic testing proved negative. She had a screening mammogram approximately 16 months prior to her presentation which revealed a group of calcifications in the upper outer quadrant of the left breast. This finding led to two diagnostic mammograms completed 6 months apart. These studies revealed a stable upper outer left breast microcalcification measuring up to 13 mm (Figure 1).

**Figure 2:**
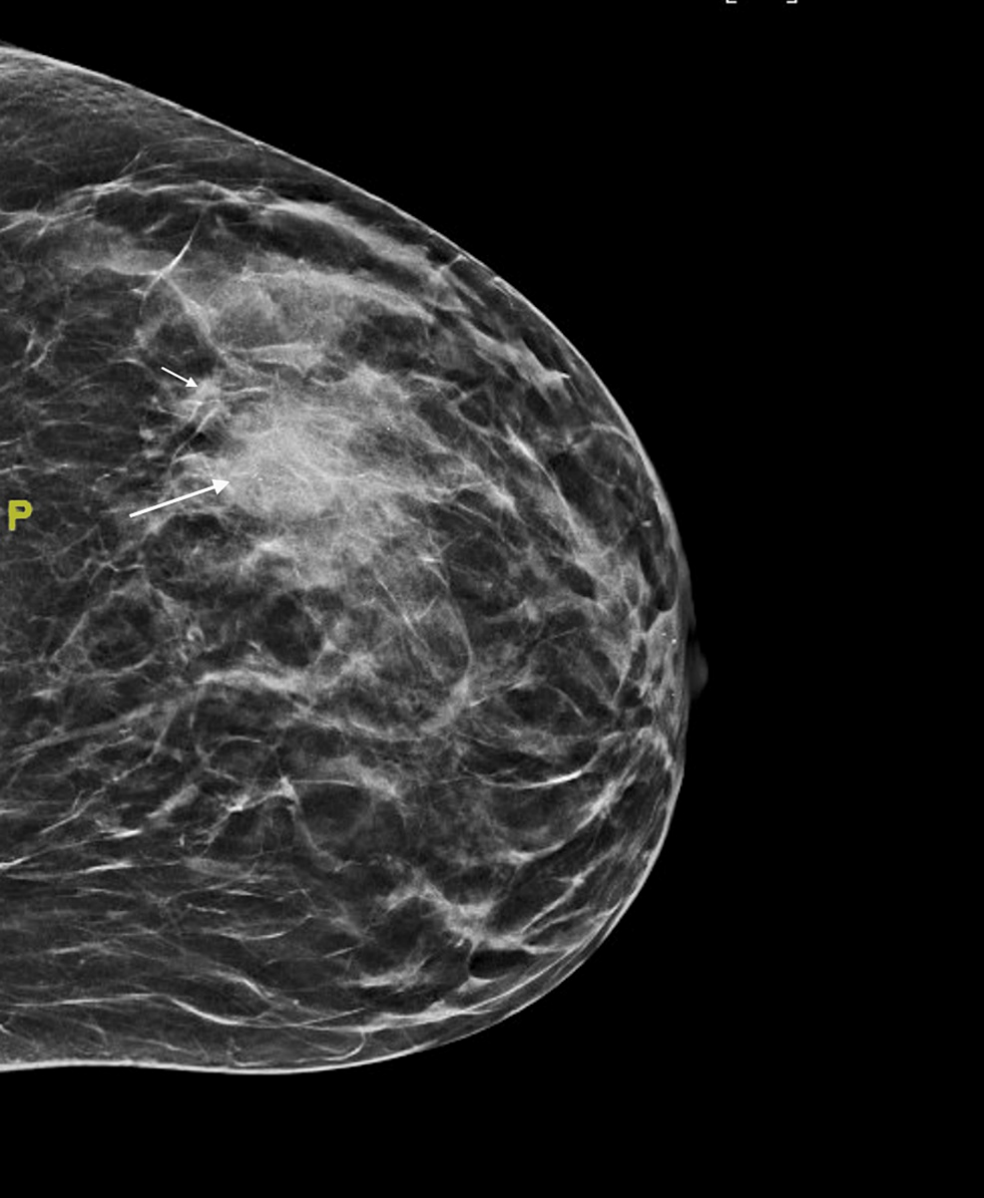
Diagnostic 3D mammogram: left craniocaudal view showing 2.4 cm circumscribed mass in the 2 o’clock position in the left breast and a smaller lateral mass measuring up to 0.5 cm.

**Figure 3:**
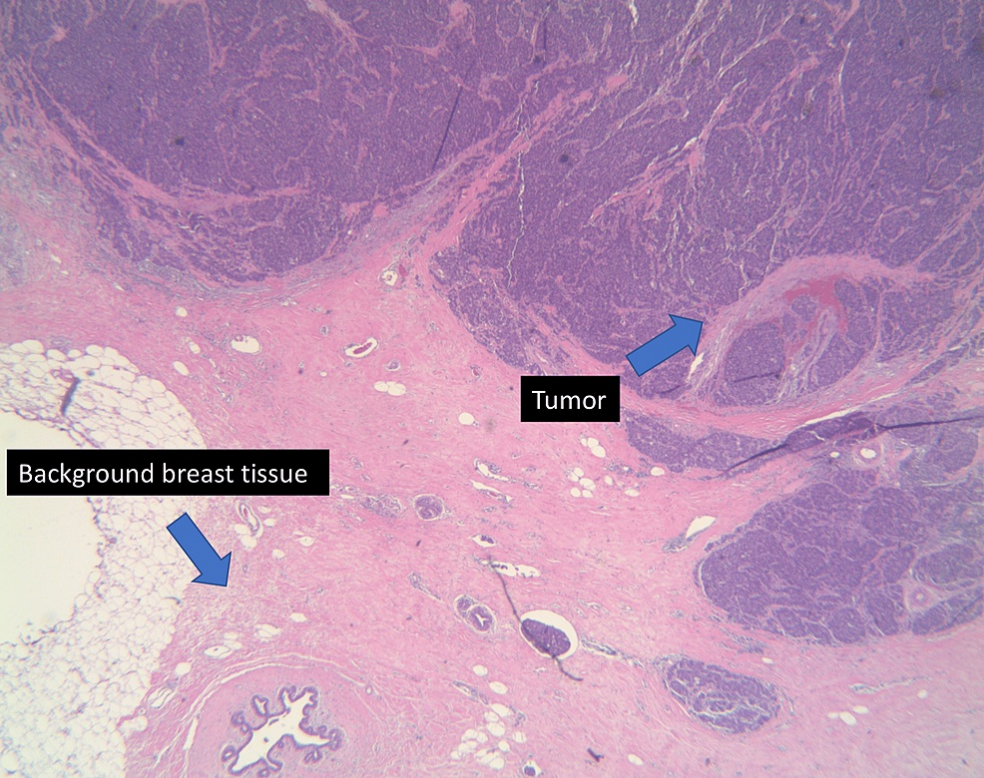
Histopathological examination of the specimen stained on low (2.5x) highlighting breast tissue involved by a large infiltrate of tumor; the findings are highlighted with arrows.

**Figure 4:**
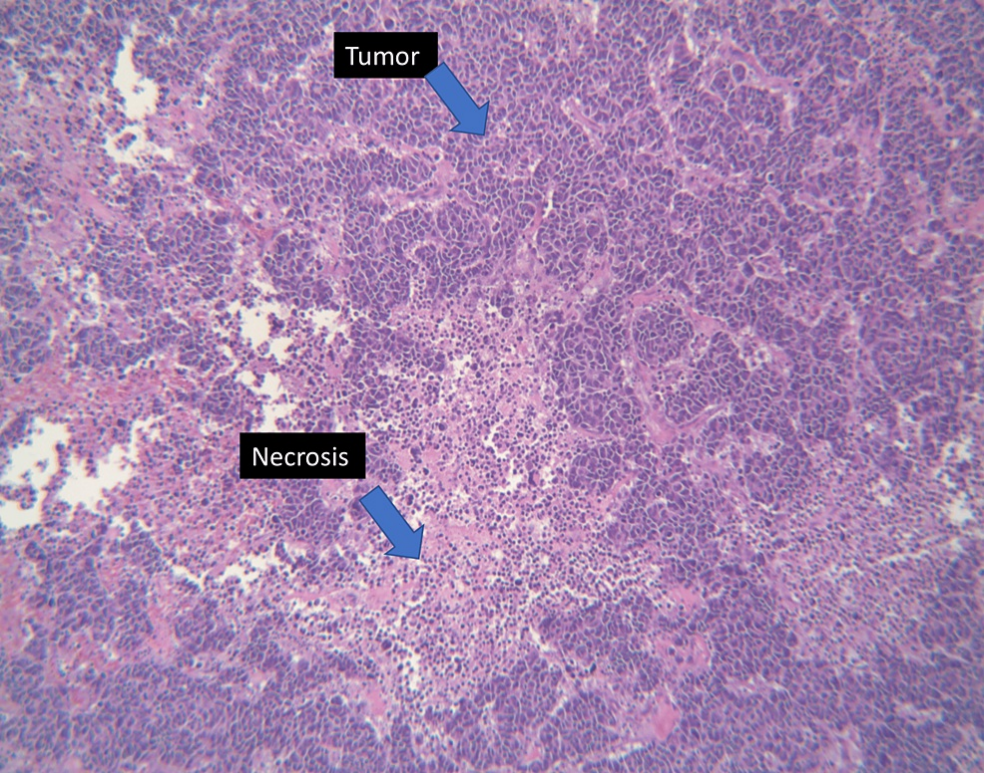
Low power field (10x) showing tumor in clusters/ribbons pattern with background necrosis; the findings are highlighted with arrows.

**Figure 5:**
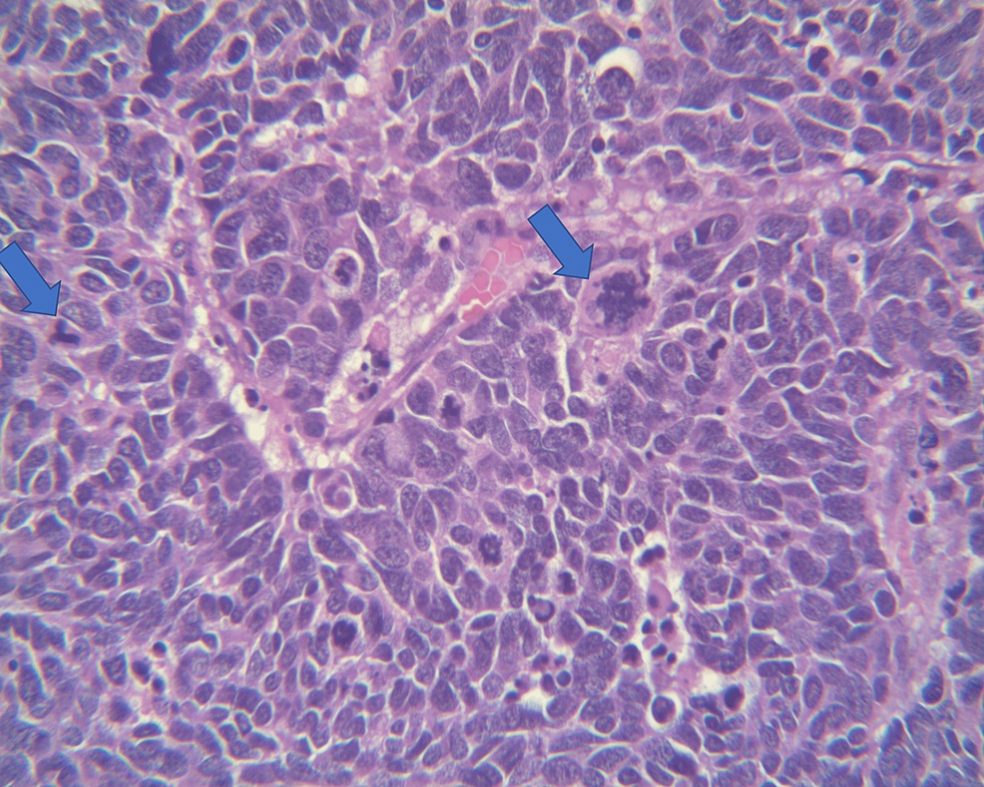
High power field (40x) showing basophilic tumor cells with finely dispersed chromatin, minimal cytoplasm, nuclear molding, smudging, high mitotic rate, and no distinct nucleoli. Mitotic figures are highlighted with arrows. Findings are consistent with the diagnosis of small cell carcinoma.

On presentation to the office, a clinical breast exam revealed a palpable, mobile 3 x 3 cm mass in the left upper outer quadrant. No axillary lymphadenopathy or right breast masses were appreciated. The most recent bilateral diagnostic 3D mammogram, completed eight months after her previous diagnostic mammogram, revealed a 2.4 cm circumscribed mass in the 2 o'clock position in the left breast and a smaller lateral mass measuring up to 0.5 cm (Figure 2). The diagnostic right mammogram was negative. The area was targeted by ultrasound, which again confirmed the presence of the lobulated mass. A core needle biopsy was performed on both masses in April 2022. The larger mass revealed small cell carcinoma. Histopathological examination of the larger mass showed breast tissue involved by a large infiltrative tumor in a clusters/ribbons pattern (Figures 3, 4). Neoplastic cells had a high nuclear-to-cytoplasmic ratio, nuclear molding, high mitotic rate, and no distinct nucleoli (Figure 5). Immunohistochemical stains were strongly positive for pancytokeratin, moderately positive for synaptophysin, and negative for chromogranin. GATA3, TTF-1, and CDX2 stains were also negative. Estrogen receptor (ER), progesterone receptor (PR), and HER2/neu stains were all negative. There was no presence of ductal carcinoma in situ (DCIS). These findings were consistent with the diagnosis of small cell carcinoma. Of note, the smaller mass revealed fibrocystic changes.

**Figure 6:**
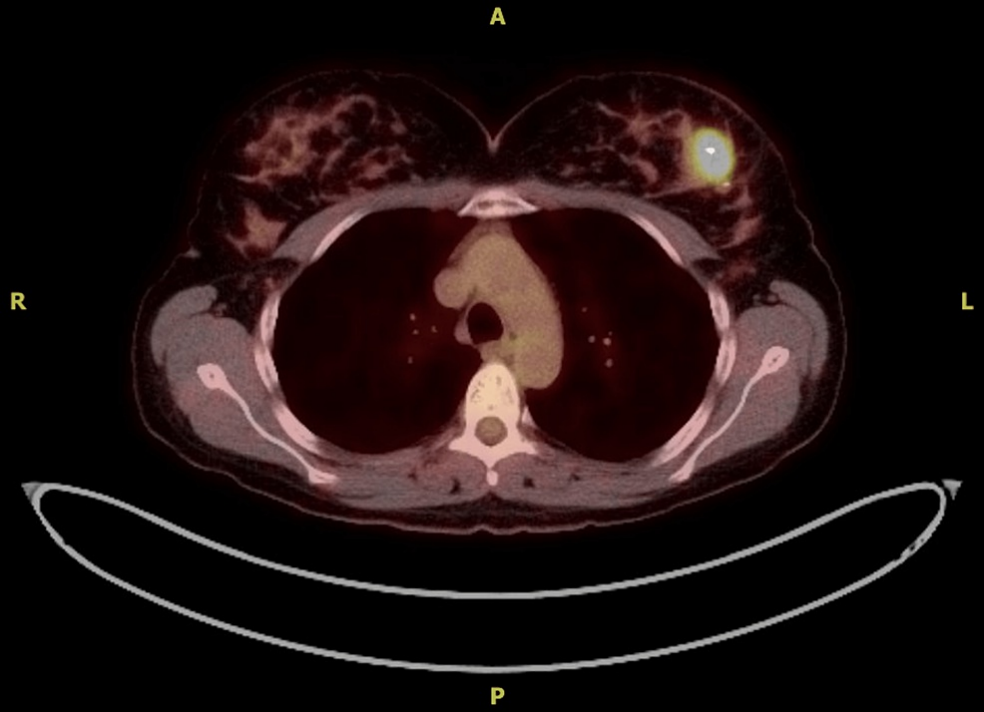
PET scan from the axial section showing the active primary lesion in the left breast measuring 2.4 cm, with a standard uptake value of 7.9. PET: positron emission tomography.

The next steps in our management involved the exclusion of metastatic disease and included several imaging studies. A positron emission tomography (PET) scan revealed a 2.4 cm soft tissue mass in the left breast with an SUV of 7.9 (Figure 6). Notably, there were a few non-enlarged lymph nodes in the left axilla with a slight increase in fluorodeoxyglucose (FDG) uptake, with a maximum value of 2.1; otherwise, no distant metastatic disease was reported. Magnetic resonance imaging (MRI) of the brain was also negative for metastatic disease. 

The patient underwent a left lumpectomy and axillary node dissection in May 2022. Pathology revealed a 25 mm small cell carcinoma with additional satellite tumor nodules and tumor involvement in the inferior margin. Two of the 16 axillary lymph nodes were positive for metastatic disease, and there was extensive angiolymphatic invasion. Immunohistochemical stains were consistent with those from the core needle biopsies. She was classified as pT2N1a. Due to the positive surgical margin and the presence of satellite tumor nodules, the patient's treatment plan included neoadjuvant chemotherapy followed by re-excision lumpectomy, radiation, and adjuvant chemotherapy. She tolerated and completed four cycles of VP-16 (etoposide) and cisplatin-based chemotherapy by August 2022. A repeat PET-CT scan performed after chemotherapy showed no abnormal uptake and resolution of the previously noted left breast mass. The patient underwent a left re-excision lumpectomy in September 2022. Pathology revealed 12 mm of residual small cell carcinoma status post-chemotherapy (ypT1cN1a). Positive tumor involvement was noted in the posterior and superior margins. One intraparenchymal lymph node was positive for metastatic disease, and angiolymphatic invasion was present. Due to the positive margins, the patient underwent a left breast simple mastectomy in October 2022. Pathology showed a 20 mm residual small cell carcinoma status post-chemotherapy and lumpectomy (pT1cN1a). All margins were negative, greater than 10 mm, and there was evidence of angiolymphatic invasion. Benign findings from the left mastectomy included changes at the biopsy cavity site, diffuse sclerotic stroma, focal benign intraductal calcifications, two fibroadenomas measuring 2-3 mm, and focal usual ductal hyperplasia. After the left breast mastectomy, the patient tolerated and completed post-mastectomy radiation therapy, 15 minutes a day for 30 treatments in December 2022.

Since there is no optimal treatment guideline defined, it was favored to manage the patient according to the tumor histology and treat as limited-stage extrapulmonary SCC. No additional systemic therapy was recommended because she had already received standard systemic therapy (four cycles of etoposide and cisplatin-based therapy), and the risk-benefit of additional adjuvant systemic therapy is unknown. There was no indication for immunotherapy. Prophylactic cranial irradiation (PCI) was not recommended by radiation oncologists, as the benefits and risks are unknown in this patient's case. The patient received a second opinion at the University of Michigan Health Breast Oncology Clinic, which concurred with her current management and plan. The patient agreed to undergo CT scans of the chest, abdomen, and pelvis every six months for surveillance imaging. She will continue to follow up with oncology every three months and with radiation oncology annually.

## Discussion

PSCCB is a very rare subtype of breast cancer, with limited information available to both providers and patients. The data that are available are largely based on case reports, such as this one, which underscores the importance of showcasing these rare presentations. PSCCB typically presents in postmenopausal women as a palpable breast mass; however, interestingly, two cases of PSCCB have been reported in males [6]. In a review of 53 cases of PSCCB, the mean age of presentation was 53 years (age range 29-81), tumor size ranged from 1-18 cm (mean 4.53), and axillary node metastasis was present in 61.7% of cases [6]. Overall, there are no remarkable differences in clinical presentation from other types of breast cancer [4].

Primary neuroendocrine tumors of the breast are rare, which is also consistent with metastatic neuroendocrine tumors, which account for only 1-2% of metastases to the breast [4]. Since PSCCB has similar histology, morphology, and immunohistochemical features to pulmonary small cell carcinoma, imaging studies are very important for diagnosing PSCCB and ruling out non-mammary primary sites. Wade et al. reported the first well-documented case of PSCCB in 1983 and claimed that the presence of regional metastases and radiographic studies that excluded other primary tumor sites strongly supported primary breast lesions [7]. Additionally, immunohistochemical stains also aid in the diagnosis of PSCCB. The most sensitive and specific markers for neuroendocrine tumors are synaptophysin and chromogranin A [4]. Less sensitive and specific markers include neuron-specific enolase and CD56. Primary breast tumors can also be supported if an in situ component is identified histologically, if there is expression of hormone receptors, or if there is expression of specific breast transcription factors (GATA3, mammaglobin, GCDFP15); however, exclusion of these factors does not rule out a breast primary [4, 8, 9]. Hormone receptor expression in PSCCB cases is found to be less prevalent when compared to well/moderately differentiated neuroendocrine carcinomas of the breast. In documented PSCCB cases, approximately 25% were either positive for ER or PR [6, 8, 9]. Her2/neu expression has only been reported in one case in the literature [10]. TTF1 and CDX2 are additional useful immunohistochemical markers. CDX2 shows positivity in 100% (5 of 5) of metastases from the gastrointestinal tract, and TTF1 shows positivity in approximately 70% (7 of 10) of metastases of the lung [4, 9]. TTF1 positivity has been observed in cases of poorly differentiated breast neuroendocrine tumors. Our case represents a good example of PSCCB in which non-mammary metastasis was excluded using PET and MRI, immunohistochemical staining was positive for synaptophysin and negative for TTF1 and CDX2. Additionally, she had regional metastasis to the lymph nodes, and her clinical course of a palpable breast mass strongly supports PSCCB.

Extrapulmonary small cell carcinoma prognosis is variable and largely depends on the stage; there is a 3-year survival rate of 28% in patients with limited disease versus 9% in patients with more extensive disease [2]. There are currently no specific guidelines for PSCCB staging, but it is suggested that they be staged similar to conventional breast cancer [4]. In a review of 53 cases of PSCCB, disease recurrence was reported in 18 cases (33.9%), with the most common sites of recurrence found in the liver, brain, lung, bone, and lymph nodes [6]. The review also had a mean follow-up of 20.75 months (range 3-60) and showed a mortality rate of 18.9%. Factors associated with poor prognosis are younger age at diagnosis, larger tumor size, and the presence of nodal metastases in four or more axillary lymph nodes [6]. These poor prognostic factors are similar to those of other types of breast cancer.

It is recommended that patients with extrapulmonary poorly differentiated neuroendocrine carcinoma follow the treatment protocols used for primary small cell lung cancer (SCLC), as the presentation is similar; both respond well to systemic therapy but tend to be aggressive, spread rapidly, and occur at distant sites [2, 4, 11]. According to the National Comprehensive Cancer Network (NCCN), the current standard treatment for SCLC stages I-III is generally concurrent chemoradiation [12, 13]. Surgery is a possible treatment option for patients with SCLC stages I-IIA, followed by adjuvant chemotherapy or concurrent radiation. Unfortunately, because SCLC is aggressive and tends to be widely disseminated at the time of initial diagnosis, surgery is often uncommon, making systemic therapy an essential component of treatment for SCLC [12, 13]. For patients with SCLC stage IV, the preferred treatment is chemoimmunotherapy with possible radiation therapy for symptomatic management [12, 13]. SCLC is more responsive to chemotherapy and radiation therapy than other cell types of lung cancer, and both of these treatment options have been shown to improve long-term survival for patients with SCLC [14]. The combination of platinum drugs (cisplatin or carboplatin) and etoposide chemotherapy agents is the most widely used standard chemotherapeutic regimen for both SCLC and extrapulmonary poorly differentiated large or small cell neuroendocrine carcinomas [11, 13, 14]. Standard treatment options for patients with SCLC also include prophylactic cranial irradiation (PCI). PCI is a low-dose radiation to the whole brain that is used to prevent the spread of lung cancer from the lungs to the brain and may improve the long-term survival of patients who have had a good response to chemoradiation therapy [13, 14].

Due to the rarity of PSCCB, there are no current guidelines for treatment. Instead, there are general recommendations based on case reports involving a combination of chemotherapy, radiation, surgery, and possible endocrine therapy based on receptor status. Therefore, proper diagnosis of PSCCB is critical to avoid unnecessary surgical and medical therapy. Since staging of PSCCB is similar to conventional breast cancer, the majority of case reports have followed conventional breast cancer treatment options, making surgical management the most commonly used form of treatment [4, 6, 15]. In a recent review of 63 known reported cases of PSCCB, the vast majority of patients were treated surgically with 39 mastectomies (some bilateral) and 18 lumpectomies. Of the six patients who did not undergo surgery, four had metastasis at diagnosis and were treated palliatively, and the other two had loco-regional disease and were treated with chemoradiation [15]. Surgery is often supported by either neoadjuvant or adjuvant chemotherapy and radiation, depending on the clinical scenario. The most commonly used chemotherapy regimens for PSCCB are FEC (5-fluorouracil, epirubicin, cyclophosphamide) and cisplatin plus etoposide [6]. Other chemotherapy regimens have included CMF (cyclophosphamide, methotrexate, and 5-fluorouracil) and CAE (cyclophosphamide, doxorubicin, etoposide) [16]. There has been no conclusion regarding the surgical management of PSCCB.

As previously discussed, the patient in this case was managed and treated as if diagnosed with limited-stage extrapulmonary SCC, which follows similar treatment protocols used for primary SCLC. Since the mass was identified at an early stage, and she did not have notable advanced or metastatic disease on imaging, she was a candidate for surgery. Due to recurrent positive margins, the patient underwent two lumpectomies, received neoadjuvant chemotherapy between procedures, and ultimately underwent a left mastectomy followed by post-mastectomy radiation therapy. The need for multiple interventions is believed to be due to the rapidly growing and aggressive histology of small cell carcinoma. Surgery has offered a hopeful approach in previous case reports, but ultimately there has been no conclusion regarding the surgical management of PSCCB. In the review of 63 known reported cases of PSCCB, only two of the reported cases had >60-month survival. The first case was a 33-year-old female treated with doxorubicin and cyclophosphamide, followed by radical mastectomy and adjuvant radiation. The second case was a 40-year-old female who was treated with doxorubicin and cyclophosphamide, followed by a carboplatin and etoposide regimen, and ultimately underwent mastectomy [15]. With no current guidelines and no official clinical trials of PSCCB, it is difficult to conclude if surgery should continue to be the main treatment for PSCCB and/or if concurrent chemoradiation is a viable nonsurgical option.

There has been one case of PSCCB published in the literature that followed SCLC treatment guidelines and included PCI in treatment management. The patient was diagnosed with high-grade neuroendocrine (small cell) carcinoma of the breast (T3N0M0). She received treatment with five cycles of cisplatin and etoposide but declined surgical intervention. This was followed by chest wall and regional lymphatic radiation. She was treated with PCI to a dose of 2600 cGy in 13 fractions. The patient has been on surveillance for eight years and is without evidence of recurrence or metastatic disease [15]. Currently, there are no other case reports of PCI being utilized in patients with PSCCB. With the brain being a main site of metastasis for PSCCB, the question lies in whether patients would benefit from PCI. However, with such small sample sizes and only anecdotal evidence from individual institutions, it is not possible to draw conclusions on its benefits and necessity.

## Conclusions

In conclusion, PSCCB is a very rare tumor subtype that primarily presents in postmenopausal women with frequent lymph node metastasis. This tumor subtype mimics its pulmonary counterpart, making imaging and immunohistochemical features important for ruling out non-mammary primary sites. Tumor size and lymph node status are important prognostic factors, but there are no clear standardized guidelines or treatments established. There are many areas in need of further research and investigation in the management and treatment of PSCCB. Specifically, this report underscores the need for standardized surgical management, data that highlight response to systemic therapy when compared to its counterpart, primary SCLC, and a closer investigation of the role PCI therapy could play in treatment. Furthermore, it is essential to have continued reporting of PSCCB cases in order to aid in further research, help establish standardized treatment protocols, and generate a larger pool of combined data.
